# Net ultrafiltration intensity and mortality in critically ill patients with fluid overload

**DOI:** 10.1186/s13054-018-2163-1

**Published:** 2018-09-24

**Authors:** Raghavan Murugan, Vikram Balakumar, Samantha J. Kerti, Priyanka Priyanka, Chung-Chou H. Chang, Gilles Clermont, Rinaldo Bellomo, Paul M. Palevsky, John A. Kellum

**Affiliations:** 1 Department of Critical Care Medicine, The Center for Critical Care Nephrology, CRISMA, University of Pittsburgh School of Medicine, Pittsburgh, PA USA; 2 Department of Critical Care Medicine, The Clinical Research, Investigation, and Systems Modeling of Acute Illness (CRISMA) Center, University of Pittsburgh School of Medicine, Pittsburgh, PA USA; 3 Department of Medicine, University of Pittsburgh School of Medicine, Pittsburgh, PA USA; 4 Department of Biostatistics, Graduate School of Public Health, University of Pittsburgh, Pittsburgh, PA USA; 5 Department of Intensive Care Medicine, The University of Melbourne, Austin Hospital, Heidelberg, VIC Australia; 6 Renal Section, Veterans Affairs Pittsburgh Healthcare System, Pittsburgh, PA USA; 7 Critical Care Medicine, and Clinical & Translational Science, University of Pittsburgh, Suite 220, Room 206, 3347 Forbes Avenue, Pittsburgh, PA 15261 USA

**Keywords:** Net ultrafiltration, Intensity, Fluid overload, Renal replacement therapy, Dialysis, Mortality

## Abstract

**Background:**

Although net ultrafiltration (UF^NET^) is frequently used for treatment of fluid overload in critically ill patients with acute kidney injury, the optimal intensity of UF^NET^ is unclear. Among critically ill patients with fluid overload receiving renal replacement therapy (RRT), we examined the association between UF^NET^ intensity and risk-adjusted 1-year mortality.

**Methods:**

We selected patients with fluid overload ≥ 5% of body weight prior to initiation of RRT from a large academic medical center ICU dataset. UF^NET^ intensity was calculated as the net volume of fluid ultrafiltered per day from initiation of either continuous or intermittent RRT until the end of ICU stay adjusted for patient hospital admission body weight. We stratified UF^NET^ as low (≤ 20 ml/kg/day), moderate (> 20 to ≤ 25 ml/kg/day) or high (> 25 ml/kg/day) intensity. We adjusted for age, sex, body mass index, race, surgery, baseline estimated glomerular filtration rate, oliguria, first RRT modality, pre-RRT fluid balance, duration of RRT, time to RRT initiation from ICU admission, APACHE III score, mechanical ventilation use, suspected sepsis, mean arterial pressure on day 1 of RRT, cumulative fluid balance during RRT and cumulative vasopressor dose during RRT. We fitted logistic regression for 1-year mortality, Gray’s survival model and propensity matching to account for indication bias.

**Results:**

Of 1075 patients, the distribution of high, moderate and low-intensity UF^NET^ groups was 40.4%, 15.2% and 44.2% and 1-year mortality was 59.4% vs 60.2% vs 69.7%, respectively (*p* = 0.003). Using logistic regression, high-intensity compared with low-intensity UF^NET^ was associated with lower mortality (adjusted odds ratio 0.61, 95% CI 0.41–0.93, *p* = 0.02). Using Gray’s model, high UF^NET^ was associated with decreased mortality up to 39 days after ICU admission (adjusted hazard ratio range 0.50–0.73). After combining low and moderate-intensity UF^NET^ groups (*n* = 258) and propensity matching with the high-intensity group (*n* = 258), UF^NET^ intensity > 25 ml/kg/day compared with ≤ 25 ml/kg/day was associated with lower mortality (57% vs 67.8%, *p* = 0.01). Findings were robust to several sensitivity analyses.

**Conclusions:**

Among critically ill patients with ≥ 5% fluid overload and receiving RRT, UF^NET^ intensity > 25 ml/kg/day compared with ≤ 20 ml/kg/day was associated with lower 1-year risk-adjusted mortality. Whether tolerating intensive UF^NET^ is just a marker for recovery or a mediator requires further research.

**Electronic supplementary material:**

The online version of this article (10.1186/s13054-018-2163-1) contains supplementary material, which is available to authorized users.

## Background

Fluid overload (FO) is a common complication of acute illness affecting more than a third of critically ill patients and approximately two-thirds of patients with acute kidney injury (AKI) requiring renal replacement therapy (RRT) [1, 2]. Several studies have documented that FO is independently associated with more than 50% mortality among patients receiving RRT [3, 4]. Observational studies suggest that fluid removal using net ultrafiltration (UF^NET^) may be associated with improved outcomes [2], and clinical and consensus guidelines recommend UF^NET^ for the treatment of FO in patients with oliguric AKI who are resistant to diuretic therapy [5, 6]. However, the optimal intensity of UF^NET^ (i.e., rate and volume of net fluid removal) in critically ill patients remains uncertain more than 70 years after the first clinical use of ultrafiltration [7].

Less intensive UF^NET^, characterized by a slower rate or smaller volume of fluid removed, may be associated with prolonged exposure to tissue and organ edema and increased morbidity and mortality [8, 9]. More intensive UF^NET^ with a faster rate or larger volume of fluid removal, however, may be associated with increased hemodynamic and cardiovascular stress [10], leading to ischemic organ injury and mortality in critically ill patients [11]. Indeed, three observational studies in outpatients with end-stage renal disease suggest that UF^NET^ intensity > 10 ml/kg/h is associated with increased overall [12–14] and cardiovascular [12] mortality.

Understanding the relationship between UF^NET^ intensity and outcome in critically ill patients is essential for two important reasons. First, if more intensive UF^NET^ is associated with lower mortality, then clinical trials could be designed to reduce the risk of death. Second, understanding the intensity–outcome relationship will aid in standardizing UF^NET^ intensity and implementing quality measures [15, 16].

In this observational study involving a large heterogeneous cohort of critically ill patients with ≥ 5% FO and receiving RRT, we examined the association between UF^NET^ intensity and its association with risk-adjusted 1-year mortality. Because the magnitude of FO is independently associated with mortality, we hypothesized that intensive UF^NET^ would be associated with lower mortality. However, our null hypothesis was that there is no difference in mortality for an intensive UF^NET^ group compared with a less intensive UF^NET^ group.

## Methods

### Data source and study population

We conducted a retrospective study using a large tertiary care academic medical center ICU database: the High-Density Intensive Care dataset, details of which have been published elsewhere (Additional file 1: S1) [1, 17, 18]. The study population included adults admitted to medical, cardiac, abdominal transplant, cardiothoracic, surgical, neurovascular, neurotrauma and trauma ICUs during July 2000 through October 2008. We included patients with AKI receiving RRT who had a cumulative fluid balance ≥ 5% prior to RRT initiation (Additional file 1: Figure S1). We extracted the daily fluid balance before and for the duration of RRT (Additional file 1: S2), hourly mean arterial pressure (MAP) and vasopressor type and dose (Additional file 1: S3) during RRT. The University of Pittsburgh’s institutional review board approved the study.

### Determination of UF^NET^ intensity

For patients receiving continuous renal replacement therapy (CRRT), we first extracted data on the total duration (in hours) of any form of CRRT (i.e., continuous venovenous hemodiafiltration (CVVHDF), continuous venovenous hemofiltration (CVVH), continuous venovenous hemodialysis (CVVHD) and slow continuous ultrafiltration (SCUF)). We then determined the UF volume produced and the amount of substitution fluids given each hour for patients receiving CVVHDF and CVVH. The UF^NET^ each hour was calculated as the difference between the UF volume and the volume of substitution fluids [19]. For patients receiving CVVHD and SCUF, UF^NET^ corresponded to the UF volume removed. We then calculated the total number of days of CRRT for each patient based on the hourly duration of CRRT and the total UF^NET^.

For patients receiving intermittent hemodialysis (IHD), we extracted the total number of IHD sessions and the UF volume removed per session from the time of ICU admission to the end of ICU stay. We excluded patients if they received IHD prior to ICU admission. UF^NET^ corresponded to the volume ultrafiltered during each session. We then expressed the total number of IHD sessions as the number of days for each patient. Subsequently, we estimated the UF^NET^ intensity using the equation:

$$ {\mathrm{UF}}^{\mathrm{NET}}\mathrm{intensity}\left(\mathrm{ml}/\mathrm{kg}/\mathrm{day}\right)=\kern0.5em \frac{\mathrm{Total}\ {\mathrm{UF}}^{\mathrm{NET}}\mathrm{volume}\ \left(\mathrm{ml}\right)}{\mathrm{Hospital}\ \mathrm{admission}\ \mathrm{weight}\ \left(\mathrm{kg}\right)\ \mathrm{X}\ \mathrm{RRT}\ \mathrm{duration}\ \left(\mathrm{days}\right)}. $$

For instance, if an 80-kg patient is on CVVH with an UF rate of 2000 ml/h and substitution fluid of 1500 ml/h, the total UF^NET^ produced is 500 ml/h (2000 – 1500 = 500 ml) or 500 × 24 = 12,000 ml/day. The total UF^NET^ produced for 5 days is 12,000 × 5 = 60,000 ml. Thus, the total UF^NET^ intensity is [60,000 / (80 × 5)] = 150 ml/kg/day. During CVVHD and IHD, the UF volume is equivalent to UF^NET^.

### Outcomes

The primary outcome was 1-year mortality from the index ICU admission and mortality data were obtained from the Social Security Death Master File [20]. We chose 1-year mortality because our prior work showed that a positive fluid balance was associated with risk of death at 1 year and use of renal replacement therapy was associated with lower risk of death in patients with a positive fluid balance [1]. Secondary outcomes included hospital length of stay, hospital mortality and renal recovery. Renal recovery was defined as alive *and* independent from RRT at 1 year. Dialysis dependence data were obtained from the US Renal Data System [21].

### Statistical analysis

We stratified UF^NET^ intensity into three groups because of the nonlinear (i.e., J-shaped) association between UF^NET^ intensity and hospital mortality (Additional file 1: Figure S2). We defined UF^NET^ ≤ 20 ml/kg/day as “low” intensity, UF^NET^ > 20 to ≤ 25 ml/kg/day as “moderate” intensity and UF^NET^ > 25 ml/kg/day as “high” intensity. Categorical variables were compared using the chi-squared test, and continuous variables using-one way analysis of variance and the Kruskal–Wallis test. We assessed time-to-mortality censored at 1 year using Kaplan–Meier failure plots.

We used three methods to examine the association between UF^NET^ intensity and mortality. First, we fitted logistic regression and estimated risk-adjusted odds ratios (AORs) for high and moderate intensity, compared with low intensity UF^NET^ (reference), on 1-year mortality. Second, we fitted Gray’s survival model [22, 23] to estimate risk-adjusted hazard ratios (AHRs) for time to mortality using four time nodes and five intervals (Additional file 1: S4). We adjusted for differences in age, sex, race, body mass index, history of liver disease and sequela from liver disease, admission for liver transplantation, admission for surgery, baseline glomerular filtration rate, Acute Physiologic and Chronic Health Evaluation (APACHE) III score, presence of sepsis, use of mechanical ventilation, percentage of FO before initiation of RRT, oliguria before initiation of RRT, time to initiation of RRT from ICU admission, MAP on first day of RRT initiation, cumulative vasopressor dose and cumulative fluid balance during RRT, first RRT modality and duration of RRT.

Third, in order to account for indication bias, we conducted a propensity score-matched analysis. Since the mortality associated with moderate (> 20 to ≤ 25 ml/kg/day) vs high (> 25 ml/kg/day) or moderate (> 20 to ≤ 25 ml/kg/day) vs low (≤ 20 ml/kg/day) intensity UF^NET^ was not different (Table 1), we combined the moderate and low-intensity groups into a single low-intensity group (reference). We then matched the low-intensity UF^NET^ (≤ 25 ml/kg/day) with the high-intensity UF^NET^ (> 25 ml/kg/day) using propensity scores on a 1:1 basis without replacement, creating 258 matched pairs (Additional file 1: S5).

**Table 1:** Baseline characteristics of study population by net ultrafiltration Intensity

	≤ 20 ml/kg/day (*n* = 475)	> 20 to ≤ 25 ml/kg/day (*n* = 166)	> 25 ml/kg/day (*n* = 434)	*p* value
Age (years), median (IQR)	61 (52–69)	59 (51–71)	58 (48–70)	0.16
Male sex	301 (63.4)	114 (68.7)	218 (50.2)	< 0.001
Race				
Caucasian	380 (80)	136 (81.9)	335 (77.2)	0.018
African-American	24 (5.1)	6 (3.6)	43 (9.9)	
Other	71 (14.9)	24 (14.5)	56 (12.9)	
BMI (kg/m^2^), median (IQR)	28.3 (24.2–34.3)	27.7 (24.2–31.7)	25.1 (21.9–29.3)	< 0.001
Comorbid condition				
Hypertension	169 (35.6)	72 (43.4)	161 (37.1)	0.19
Diabetes	121 (25.5)	34 (20.5)	97 (22.4)	0.33
Cardiac disease	84 (17.7)	36 (21.7)	99 (22.8)	0.14
Heart failure	70 (14.7)	30 (18.1)	86 (19.8)	0.12
Vascular disease	41 (8.6)	16 (9.6)	43 (9.9)	0.79
Liver disease	164 (34.5)	47 (28.3)	107 (24.7)	0.005
Sequela from liver disease	137 (28.8)	43 (25.9)	95 (21.9)	0.056
Malignancy	23 (4.8)	4 (2.4)	14 (3.2)	0.26
Liver transplantation	43 (9.1)	13 (7.8)	42 (9.7)	0.77
Multiple comorbidity	298 (62.7)	93 (56)	252 (58.1)	0.19
Surgical admission	321 (67.6)	122 (73.5)	301 (69.4)	0.72
Medical admission	131 (27.6)	37 (22.3)	112 (25.8)	0.72
Admission for liver transplantation	102 (21.5)	31 (18.7)	53 (12.2)	0.001
Baseline serum creatinine (mg/dl), median (IQR)	1.029 (0.81–1.27)	1.035 (0.83–1.3)	1.032 (0.8–1.3)	0.89
Baseline eGFR (ml/min/1.73 m^2^)				
> 90	107 (22.5)	27 (16.3)	91 (20.9)	0.54
60–90	235 (49.5)	97 (58.4)	212 (48.9)	
30–60	89 (18.7)	30 (18.1)	92 (21.2)	
15–30	34 (7.2)	8 (4.8)	31 (7.1)	
< 15	10 (2.1)	4 (2.4)	8 (1.8)	
APACHE III score, median (IQR)^a^	95 (70–118)	91 (71–116)	91 (69–112)	0.27
Sepsis^a^	128 (26.9)	39 (23.5)	138 (31.8)	0.08
Mechanical ventilation^a^	353 (74.3)	129 (77.7)	329 (75.8)	0.66
Vasopressor^a^	261 (54.9)	87 (52.4)	218 (50.2)	0.36
Oliguria before initiation of RRT^b^				
Stage 2	50 (10.5)	9 (5.4)	21 (4.8)	0.017
Stage 3	406 (85.5)	154 (92.8)	402 (92.6)	
MAP during RRT (mmHg), mean (SD)^c^				
All patients	75.1 (0.58)	77.5 (1.19)	79.4 (0.62)	< 0.001
CRRT only (*n* = 386)	72.7 (0.70)	72.4 (1.89)	77.5 (1.01)	< 0.001
IHD only (*n* = 210)	85 (1.84)	84.1 (2.85)	82.1 (1.27)	0.77
CRRT and IHD (*n* = 487)	74.5 (0.91)	79.1 (1.66)	79.7 (0.98)	0.002
Vasopressor dose (NE), median (IQR)^c,d^				
All patients	0.11 (0.04–0.25)	0.09 (0.03–0.21)	0.09 (0.04–0.25)	0.25
Patients on CRRT only	0.14 (0.05–0.30)	0.13 (0.03–0.25)	0.10 (0.03–0.28)	0.31
Patients on IHD only	0.01 (0.01–0.03)	0.06 (0.01–0.11)	0.03 (0.01–0.07)	0.67
Patients on both CRRT and IHD	0.08 (0.03–0.16)	0.08 (0.02–0.16)	0.07 (0.03–0.19)	0.85

Data presented as *n* (%) unless stated otherwise

*IQR* interquartile range, *BMI* body mass index, *eGFR* estimated glomerular filtration, *APACHE* Acute Physiology and Chronic Health Evaluation, *RRT* renal replacement therapy, *MAP* mean arterial pressure, *SD* standard deviation, *CRRT* continuous renal replacement therapy, *IHD* intermittent hemodialysis, *NE* norepinephrine equivalents

^a^At intensive care unit admission

^b^Patients were classified to have developed oliguria according to the maximum Kidney Disease Improving Global Outcome criteria based on urine output [5]

^c^On the day 1 of RRT

^d^All vasopressors were standardized in terms of NE (Additional file 1: S3) [30–32]

We performed five sensitivity analyses and two subgroup analyses. First, we restricted the UF^NET^ intensity only up to 72 h from initiation of RRT. Second, we used an alternative definition of UF^NET^ intensity moving the threshold down as follows: low, < 15 ml/kg/day; moderate, 15–20 ml/kg/day; and high, > 20 ml/kg/day. Third, we moved the threshold up: low, < 25 ml/kg/day; moderate, 25–30 ml/kg/day; and high, > 30 ml/kg/day. Fourth, we divided the cohort into tertiles: low, ≤ 16.7 ml/kg/day; moderate, 16.7 to ≤ 27.7 ml/kg/day; and high, > 27.7 ml/kg/day. Fifth, we performed quantitative bias analysis to assess the magnitude of a hypothetical unmeasured confounder that would be necessary to account for the association between UF^NET^ intensity and risk-adjusted mortality (Additional file 1: S6) [24, 25].

Sixth, we restricted our analyses only to the subgroup of patients with > 20% FO. Seventh, we confined our analysis of UF^NET^ intensity to the hour (i.e., ml/kg/h) instead of the day among the subgroup of patients who only received CRRT as follows: low, < 0.5 ml/kg/h; moderate, 0.5–1.0 ml/kg/h; and high, > 1 ml/kg/h. Statistical analyses were performed using SAS 9.3 (SAS Institute, Cary, NC, USA), Gray’s model used R 3.2.1, and quantitative bias analysis was performed using STATA 15 (STATCorp., TX, USA). All hypotheses tests were two-sided with a significance level of *p* < 0.05.

## Results

### Study population and patient characteristics

Of 45,568 patients, we excluded patients with no available baseline weight (*n* = 2214), ICU duration ≤ 48 h (*n* = 18,032), death within 72 h of ICU admission (*n* = 663), chronic dialysis (*n* = 2386), admission for or with history of renal transplantation (*n* = 1232), serum creatinine ≥ 3.5 mg/dl within 1 year of hospitalization (*n* = 147) and missing data on fluid balance (*n* = 2810). Of 18,084 patients in whom cumulative fluid balance data were available, we excluded those with cumulative fluid balance < 5% of body weight (*n* = 9900). Of patients with cumulative balance ≥ 5% of body weight (*n* = 8184), we excluded those who did not receive RRT (*n* = 7023) and patients without data on UF^NET^ (*n* = 86) (Additional file 1: Figure S1).

Of 1075 patients, the distribution of low, moderate and high-intensity UF^NET^ groups was 44.2%, 15.2% and 40.4%, respectively. Minor differences were noted among male sex, race and body mass index between the groups (Table 1). There was a higher prevalence of liver disease (34.5%), sequela from liver disease (28.8%) and liver transplantation (21.5%) among those with low-intensity UF^NET^. There was a higher prevalence of oliguria in those who received moderate and high-intensity UF^NET^. Patients in the low-intensity UF^NET^ group had lower MAP compared with the moderate and high-intensity UF^NET^ groups (Table 1 and Additional file 1: Table S1).

Cumulative FO before RRT initiation was lowest in the low-intensity group, compared with the moderate and high-intensity UF^NET^ groups (15.6% vs 17.3% vs 21% of body weight, respectively, *p* < 0.001; Table 2). Following initiation of RRT, the median cumulative FB for the low, medium and high-intensity UF^NET^ groups was 13.5 vs 22 vs 19 l, *p* < 0.001; Table 2). During RRT, the MAP was lower and the cumulative vasopressor dose was higher in the low-intensity UF^NET^ group compared with the moderate and high-intensity UF^NET^ groups (Table 2 and Additional file 1: Table S1).

**Table 2:** Fluid balance, RRT characteristics and outcomes by intensity of net ultrafiltration

	≤ 20 ml/kg/day (*n* = 475)	> 20 to ≤ 25 ml/kg/day (*n* = 166)	> 25 ml/kg/day (*n* = 434)	*p* value
Fluids administered in the first 24 h of ICU admission (L), median (IQR)	5.3 (3.5–7.9)	5.1 (3.6– 7.8)	5.23 (3.3–8.1)	0.88
Fluid balance after ICU admission (L), median (IQR)				
At 72 h	7.9 (4.4–12)	7.8 (4.7–13.3)	7.6 (4.7–11.6)	0.71
At 7 days	10.1 (6.7–15.2)	10.5 (6.4–15.7)	10.1 (6.4–15.1)	0.78
Average before RRT	2.3 (1.2–4.4)	2.7 (1.5–4.3)	2.3 (1.2–4.2)	0.33
Cumulative before RRT (%)	15.6 (10–25)	17.3 (9.9–28.6)	21 (12.4–33.7)	< 0.001
Duration from ICU admission to RRT (days), median (IQR)	7 (2–13)	5 (3–12)	6 (3–16)	0.27
RRT duration (days), median (IQR)	4.7 (1.5–11.7)	8.7 (4.5–16.7)	7 (3.1–12.7)	< 0.001
Cumulative FB excluding UF^NET^ for duration of RRT (L), median (IQR)^a^	13.5 (4.2–32.8)	22 (8.9–45.1)	19 (7.3–37.2)	< 0.001
MAP for duration of RRT (mmHg), mean (SD)	75.2 (0.6)	77.4 (0.8)	80.1 (0.53)	< 0.001
Cumulative vasopressor dose for duration of RRT (NE), median (IQR)^a^	15.7 (4.3–38.6)	11.4 (1.2–34.7)	8.1 (0.9–25.7)	< 0.001
First RRT modality				
IHD	121 (25.5)	52 (31.3)	127 (29.3)	0.25
CRRT	354 (74.5)	114 (68.7)	307 (70.7)	
CRRT duration (days), median (IQR)	3.9 (1.5–7.7)	5.8 (3.6–9.4)	5.9 (2.8–9.5)	< 0.001
UF^NET^ volume during CRRT (L), median (IQR)	3.4 (0.9–10.2)	11.6 (5.4–19.2)	16.2 (7.5–28.4)	< 0.001
IHD duration (days), median (IQR)	2 (5–9)	7 (3–13)	4 (2–8)	0.004
UF^NET^ volume during IHD (L), median (IQR)	5.5 (2.2–13.5)	12.6 (4.4–19.7)	9.2 (4–17.2)	< 0.001
Both CRRT and IHD duration (days), median (IQR)	14.7 (9.7–22.9)	15.2 (9.2–21.9)	10.7 (6.9–18.4)	< 0.001
UF^NET^ volume during CRRT and IHD (L), median (IQR)	19.5 (9.5–33.9)	27.9 (18.5–42.1)	26.6 (17.8–46.1)	< 0.001
Hospital length of stay (days), median (IQR)	32 (17–54)	37.5 (23–65)	37 (23–61)	< 0.001
Hospital mortality	272 (57.3)	70 (42.2)	187 (43.1)	< 0.001
1-year mortality	331 (69.7)	100 (60.2)	258 (59.4)	0.003
Renal recovery at 1 year^b^	119 (25.1)	48 (28.9)	138 (31.8)	0.078
Renal recovery at 1 year in survivors^b^	119 (82.6)	48 (72.7)	138 (78.4)	0.25

Data presented as *n* (%) unless stated otherwise

*RRT* renal replacement therapy, *ICU* intensive care unit, *IQR* interquartile range, *FB* fluid balance, *UF*^*NET*^ net ultrafiltration, *MAP* mean arterial pressure, *SD* standard deviation, *NE* norepinephrine equivalents, *IHD* intermittent hemodialysis, *CRRT* continuous renal replacement therapy

^a^All vasopressors were standardized in terms of NE (Additional file 1: S3) [30–32]

^b^Renal recovery was defined as alive and independent of RRT at 1 year

The median duration of RRT for the low, moderate and high-intensity UF^NET^ groups was 4.7 vs 8.7 vs 7 days, respectively (*p* < 0.001). The median duration of CRRT was 3.9 vs 5.8 vs 5.9 days (*p* < 0.001) and the median UF^NET^ volume was 3.4 vs 11.6 vs 16.2 L (*p* < 0.001). The median duration of IHD was 2 vs 7 vs 4 days (*p* = 0.004) and the median UF^NET^ volume was 5.5 vs 12.6 vs 9.2 L (*p* < 0.001). The median duration of RRT for patients who received both CRRT and IHD was 14.7 vs 15.2 vs 10.7 days (*p* < 0.001) and the median UF^NET^ volume was 19.5 vs 27.9 vs 26.6 L (*p* < 0.001). The median hospital length of stay was 32 vs 37.5 vs 37 days (*p* < 0.001) (Table 2). This shorter length of stay among patients with low-intensity UF^NET^ was primarily due to higher mortality in this group. However, there was no difference in renal recovery at 1 year (25.1% vs 28.9% vs 31.8%, *p* = 0.078) as well as within the subgroup of survivors at 1 year (82.6% vs 72.7% vs 78.4%, *p* = 0.25) between the three groups.

### Association between UF^NET^ intensity and mortality

**Fig. 1:**
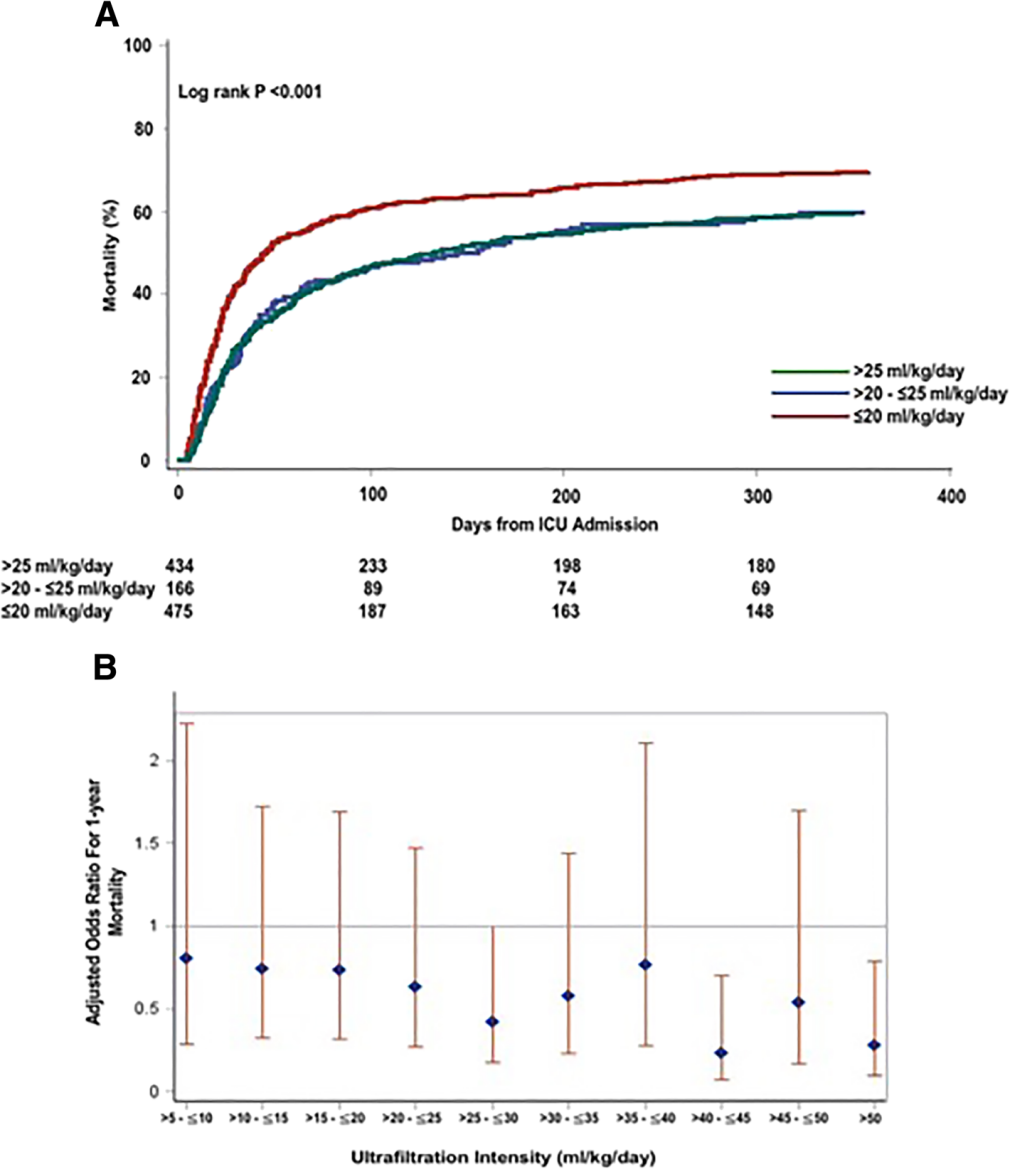
**a**. Association between net ultrafiltration intensity and time to mortality. Kaplan–Meier failure plots by UF^NET^ intensity for probability of death over 1 year from ICU admission in overall cohort (*n* = 1075). Red line, low-intensity UF^NET^ (≤ 20 ml/kg/day); blue line, moderate-intensity UF^NET^ (> 20 to ≤ 25 ml/kg/day); green line, high-intensity UF^NET^ (> 25 ml/kg/day). Probability of death highest in low-intensity compared with moderate and high-intensity UF^NET^ groups (log-rank *p* < 0.001). **b**. Association between net ultrafiltration intensity and risk-adjusted 1-year mortality. Shown are adjusted odds ratio with 95% CI for association between UF^NET^ intensity and mortality. Increasing UF^NET^ intensity associated with trend toward lower mortality. Odds ratios adjusted for differences in age, sex, race, BMI, history of liver disease and sequela from liver disease, admission for liver transplantation, admission for surgery, baseline glomerular filtration rate, Acute Physiology and Chronic Health Evaluation III score, presence of sepsis, use of mechanical ventilation, percentage of cumulative fluid overload before initiation of RRT, oliguria before initiation of RRT, time to initiation of RRT from ICU admission, MAP on first day of RRT initiation, cumulative vasopressor dose and cumulative fluid balance during RRT, first RRT modality and duration of RRT. ICU intensive care unit

The crude hospital and 1-year mortality was higher among the low-intensity group compared with the moderate and high-intensity UF^NET^ groups: 69.7% vs 60.2% vs 59.4% (*p* = 0.003), respectively (Table 2, Fig. 1a). Using logistic regression, high-intensity compared with low-intensity UF^NET^ was associated with lower 1-year mortality (AOR 0.61, 95% CI 0.41–0.93, *p* = 0.02, *C*-statistic 0.811; Table 3 and Additional file 1: Table S2). This association persisted using UF^NET^ as a continuous variable (AOR 0.98, 95% CI 0.97–0.99, *p* = 0.005; Additional file 1: Table S3). Compared with UF^NET^ of 0–5 ml/kg/day, increasing UF^NET^ intensity was associated with a trend toward lower odds of death (*C*-statistic – 0.813; Fig. 1b), whereas moderate-intensity compared with low-intensity UF^NET^ was not associated with mortality (AOR 0.81, 95% CI 0.48–1.35, *p* = 0.41; Additional file 1: Table S2).

**Table 3:** Association between UF^NET^ intensity and 1-year risk-adjusted mortality

Covariates	Unadjusted odds ratio (95% CI)	*p* value	Adjusted^a^ odds ratio (95% CI)	*p* value
Moderate vs low-intensity UF^NET^ (reference)	0.65 (0.42–0.94)	0.024	0.81 (0.48–1.35)	0.41
High vs low-intensity UF^NET^ (reference)	0.64 (0.49–0.85)	0.002	0.61 (0.41–0.93)	0.02

*UF*^*NET*^ net ultrafiltration, *CI* confidence interval, FO fluid overload, *RRT* renal replacement therapy, *ICU* intensive care unit

^a^Adjusted for age, sex, race, body mass index, history of liver disease and sequela from liver disease, admission for liver transplantation, admission for surgery, baseline glomerular filtration rate, Acute Physiology and Chronic Health Evaluation III score, presence of sepsis, use of mechanical ventilation, percentage of FO before initiation of RRT, oliguria before initiation of RRT, time to initiation of RRT from ICU admission, mean arterial pressure on first day of RRT initiation, cumulative vasopressor dose and cumulative fluid balance during RRT, first RRT modality and duration of RRT

Using Gray’s model, high-intensity compared with low-intensity UF^NET^ had variable association with mortality. Early on after ICU admission, high-intensity UF^NET^ was associated with lowest risk of death that was subsequently attenuated over time, but nevertheless persisted up to 39 days after ICU admission (AHR range 0.50–0.73, *p* < 0.001; Table 4 and Additional file 1: Figure S3A). Subsequently, between 39 and 365 days, high-intensity UF^NET^ was not associated with mortality (AHR range 0.76–1.02). High-intensity compared with moderate-intensity UF^NET^ was only associated with lower risk of death up to 15 days (AHR 0.53, 95% CI 0.33–0.86; Additional file 1: Table S5 and Figure S3C).

**Table 4:** Association between intensity of net ultrafiltration and time to mortality from Gray’s model

Characteristic	Adjusted hazard ratio (95% CI) by time interval^a^					*p* value
	5–15 days	15–23 days	23–39 days	39–91 days	91–365 days	
High vs low UF^NET^	0.50 (0.35–0.71)	0.62 (0.46–0.82)	0.73 (0.55–0.97)	0.76 (0.56–1.04)	1.02 (0.71–1.47)	< 0.001
High vs moderate UF^NET^	0.53 (0.33–0.86)	0.69 (0.46–1.02)	0.75 (0.52–1.09)	0.77 (0.518–1.142)	1.16 (0.72–1.85)	0.039
Moderate vs low UF^NET^	0.98 (0.62–1.57)	0.87 (0.59–1.27)	0.996 (0.69–1.43)	1.01 (0.69–1.47)	0.844 (0.53–1.34)	0.91

Shown are adjusted hazard ratios estimated from Gray’s model for association between intensity of UF^NET^ and mortality for each time interval. Models included five time intervals and four time nodes with the default timing of nodes chosen by the statistical program based on the number of observations within each time interval. Hazard ratio < 1 suggests that UF^NET^ intensity is associated with lower mortality, and hazard ratio > 1 suggests UF^NET^ intensity is associated with higher mortality. *p* values reported are for the ranges of hazard ratios from the model

*CI* confidence interval, *UF*^*NET*^ net ultrafiltration, FO fluid overload, *RRT* renal replacement therapy, *ICU* intensive care unit

^a^Adjusted for age, sex, race, body mass index, history of liver disease and sequela from liver disease, admission for liver transplantation, admission for surgery, baseline glomerular filtration rate, Acute Physiology and Chronic Health Evaluation III score, presence of sepsis, use of mechanical ventilation, percentage of FO before initiation of RRT, oliguria before initiation of RRT, time to initiation of RRT from ICU admission, mean arterial pressure on first day of RRT initiation, cumulative vasopressor dose and cumulative fluid balance during RRT, first RRT modality and duration of RRT

**Fig. 2:**
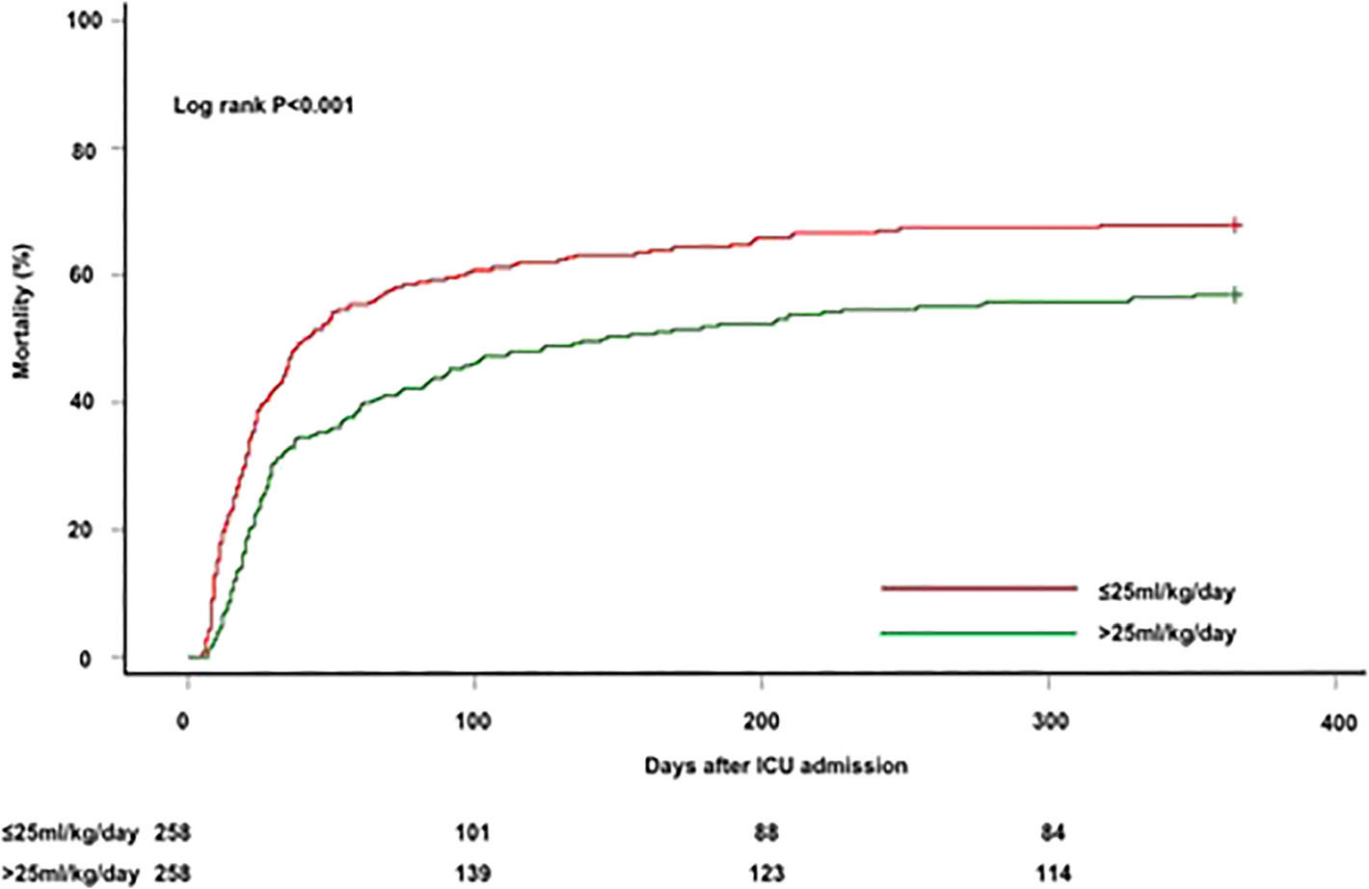
Association between net ultrafiltration intensity and time to mortality in propensity-matched cohort. Kaplan–Meier failure plots by UF^NET^ for probability of death over 1 year from ICU admission among patients with UF^NET^ ≤ 25 ml/kg/day (*n* = 258) compared with propensity-matched patients with UF^NET^ > 25 ml/kg/day (*n* = 258). Red line, UF^NET^ ≤ 25 ml/kg/day; green line, UF^NET^ > 25 ml/kg/day. Probability of death lower among patients who received UF^NET^ > 25 ml/kg/day compared with UF^NET^ ≤ 25 ml/kg/day (log-rank *p* < 0.001). ICU intensive care unit

After propensity matching, 258 matched pairs were created wherein patients with UF^NET^ intensity ≤ 25 ml/kg/day had similar baseline characteristics compared with UF^NET^ intensity > 25 ml/kg/day, except for cumulative vasopressor dose (Additional file 1: Table S4). Patients with UF^NET^ intensity > 25 ml/kg/day compared with ≤ 25 ml/kg/day had lower 1-year mortality (57% vs 67.8%, *p* = 0.01; Fig. 2), which persisted after adjusting for vasopressor dose (AOR 0.63, 95% CI 0.44–0.90, *p* = 0.011).

### Sensitivity analyses

When UF^NET^ intensity calculation was limited within 72 h of initiation of RRT, high-intensity UF^NET^ was associated with lower mortality (AOR 0.56, 95% CI 0.35–0.88, *p* = 0.013; Table 5). Using the alternative thresholds of low, moderate and high-intensity UF^NET^ of < 15 ml/kg/h, 15–20 ml/kg/h and > 20 ml/kg/h, respectively, we found UF^NET^ intensity > 20 ml/kg/h was associated with lower mortality (AOR 0.63, 95% CI 0.41–0.97, *p* = 0.038). Similar results were found moving the threshold up (AOR 0.58, 95% CI 0.34–0.99, *p* = 0.04; Table 5) and using tertile cutoff values (AOR 0.61, 95% CI 0.39–0.97, *p* = 0.037; Table 5). The quantitative bias analysis indicated that our results would be robust unless an unmeasured confounder was at least twice as prevalent among patients who received high-intensity UF^NET^ as among those with low-intensity UF^NET^ (Additional file 1: Figure S4). The unmeasured confounder should have an OR < 0.7 (i.e., reduced risk of death by more than 30%) to mask a null association between high-intensity UF^NET^ and risk-adjusted mortality (Additional file 1: S6 and Figure S4).

**Table 5:** Sensitivity and subgroup analyses of net ultrafiltration intensity and mortality

Characteristic	Net ultrafiltration intensity	Adjusted odds ratio (95% CI)^a^	*p* value
Sensitivity analysis			
UF^NET^ up to 72 h after RRT initiation^b^	High vs low	0.56 (0.35–0.88)	0.013
	Moderate vs low	1.10 (0.58–2.11)	0.76
Alternative UF^NET^ threshold^c^	High vs low	0.63 (0.41–0.97)	0.038
	Moderate vs low	0.91 (0.53–1.58)	0.74
Alternative UF^NET^ threshold^d^	High vs low	0.58 (0.34–0.99)	0.044
	Moderate vs low	0.66 (0.43–1.01)	0.053
Alternative UF^NET^ threshold^e^	High vs low	0.61 (0.39–0.97)	0.0371
	Moderate vs low	0.69 (0.45–1.07)	0.096
Subgroup analysis			
UF^NET^ among subgroup of patients with cumulative FB > 20% before RRT^f^	High vs low	0.52 (0.26–1.05)	0.07
	Moderate vs low	0.74 (0.29–1.84)	0.51
Alternative UF^NET^ threshold among subgroup of patients who only received CRRT^g^	High vs low	0.41 (0.24–0.71)	0.0013
	Moderate vs low	0.68 (0.39–1.18)	0.17

*CI* confidence interval, *UF*^*NET*^ net ultrafiltration, *RRT* renal replacement therapy, *FB* fluid balance, *CRRT* continuous renal replacement therapy, FO fluid overload, *ICU* intensive care unit

^a^Adjusted for differences in age, sex, race, body mass index, history of liver disease and sequela from liver disease, admission for liver transplantation, admission for surgery, baseline glomerular filtration rate, Acute Physiology and Chronic Health Evaluation III score, presence of sepsis, use of mechanical ventilation, percentage of FO before initiation of RRT, oliguria before initiation of RRT, time to initiation of RRT from ICU admission, mean arterial pressure on first day of RRT initiation, cumulative vasopressor dose and fluid balance during RRT, first RRT modality and duration of RRT

^b^UF^NET^ intensity calculated using RRT duration of 72 h as a cutoff value in 1075 patients

^c^Threshold for low, moderate and high UF^NET^ varied as follows in 1075 patients: low, < 15 ml/kg/day; moderate, 15–20 ml/kg/day; and high, > 20 ml/kg/day

^d^Threshold for low, moderate and high UF^NET^ varied as follows in 1075 patients: low, < 25 ml/kg/day; moderate, 25–30 ml/kg/day; and high, > 30 ml/kg/day

^e^Threshold for low, moderate and high UF^NET^ based on stratifying the cohort of 1075 patients into tertiles: low, ≤ 16.7 ml/kg/day; moderate, 16.7 to ≤ 27.7 ml/kg/day; and high, > 27.7 ml/kg/day

^f^UF^NET^ calculated within subgroup of 465 patients with cumulative FB > 20% before RRT initiation

^g^Threshold for low, moderate and high UF^NET^ varied among subgroup of 487 patients who only received CRRT as follows: low, < 0.5 ml/kg/h; moderate, 0.5–1.0 ml/kg/h; and high, > 1 ml/kg/h

### Subgroup analyses

High-intensity UF^NET^ was associated with a trend toward lower mortality among patients with > 20% FO (AOR 0.52, 95% CI 0.26–1.05, *p* = 0.07; Table 5). For patients receiving CRRT, UF^NET^ intensity > 1.0 ml/kg/h compared with UF^NET^ intensity < 0.5 ml/kg/h was associated with lower odds of death (AOR 0.41, 95% CI 0.24–0.71, *p* = 0.0013).

## Discussion

We found that UF^NET^ intensity > 25 ml/kg/day, compared with < 20 ml/kg/day, was independently associated with lower risk-adjusted 1-year mortality in critically ill patients with FO. Using Gray’s model, this survival benefit was greater early on after ICU admission and persisted up to 39 days. In the propensity-matched analysis, UF^NET^ > 25 ml/kg/day, compared with ≤ 25 ml/kg/day, was also associated with lower risk of death. To our knowledge, this is the first study in the literature examining the association between UF^NET^ intensity and long-term mortality.

Our finding is somewhat analogous to the association between intensity of solute control and mortality in critically ill patients receiving RRT in which a threshold intensity of at least 20–25 ml/kg/h of effluent dosing in CRRT or KT/V of 1.2–1.4 per session in patients receiving IHD is associated with improved survival [26, 27]. However, in contrast to studies on solute control, the optimal “dosing” for UF^NET^ in critically ill patients with fluid overload is unclear. In our study, we first explored whether there was an association between UF^NET^ dose and mortality, and then aimed to determine the overall “average dose” that is associated with a long-term mortality benefit. It is important to note that our finding does not suggest that UF^NET^ should be dosed > 25 ml/kg/day throughout the duration of fluid removal. Day-to-day dosing may vary in patients depending on the severity of fluid overload, patient tolerability and hemodynamics.

In our study only 40% of patients received intensive UF^NET^, whereas 44% of patients received less intensive UF^NET^ that has implications for care. Unlike a prescription for solute clearance, the concept of a minimum or adequate “dose” for volume clearance is not usually considered in clinical practice. Although patients who received less intensive UF^NET^ were hemodynamically unstable in our study, our findings persisted after accounting for hemodynamics, vasopressor dose and severity of illness, suggesting that less intensive UF^NET^ per se might be associated with mortality. These findings may suggest that failure to tolerate UF^NET^ > 25 ml/kg/day may portend a poor prognosis and, conversely, tolerating UF^NET^ > 25 ml/kg/day may be a predictor of recovery and lower mortality in critically ill patients with fluid overload.

Our study addresses an important knowledge gap not addressed by prior studies. While numerous studies have documented an association between the severity of FO and incremental risk of death [3, 4], none examined the UF^NET^ intensity–mortality relationship. Using the Program to Improve Care in Acute Renal Disease (PICARD) study, Bouchard et al. [4] found that patients in whom FO was corrected during RRT had lower mortality than those who remained fluid overloaded despite RRT. Using the Randomized Evaluation of Normal versus Augmented Level of Renal Replacement Therapy (RENAL RRT) cohort, Bellomo et al. [2] found that a negative fluid balance during RRT was associated with a mortality benefit. However, we asked a different question: does UF^NET^ intensity and a threshold “dose” of UF^NET^ matter in the treatment of FO independent of fluid balance?

There may be several biologic explanations for the association between UF^NET^ intensity and outcome. First, intensive UF^NET^ may reduce prolonged exposure to FO and modify host response, and could reduce the incidence of subsequent organ dysfunction [28]. Second, the salutary effects of intensive UF^NET^ may be mediated through unknown marker clearance independent of fluid balance since the association persisted despite controlling for cumulative fluid balance. Third, clinicians who decide to initiate intensive UF^NET^ may select for a unique group of patients to monitor and carefully titrate fluid removal. Fourth, clinicians and nurses may also have a broad variation in how they prescribe and/or practice UF^NET^ in the real world, which may be associated with differences in outcomes [29].

The strengths of our study was that it was robust to three different methods of sensitivity analysis. We accounted for confounding due to severity of illness, hemodynamics, vasopressor dose and cumulative fluid balance before and during RRT. Using Gray’s model, we found that high-intensity UF^NET^ was associated with survival only up to 39 days after ICU admission. This finding is in contrast with the logistic model and propensity-matched analyses, which showed mortality benefit up to 1 year. This discordant finding is due to the differences in the models that were used. In Gray’s model, the number of events between high-intensity and low-intensity UF^NET^ groups was not different within the time interval of 39–365 days. Using the logistic regression model, however, a lower odds of cumulative deaths occurred by 1 year in the high-intensity UF^NET^ group compared with the low-intensity UF^NET^ group.

Our study is not without limitations. First, given the observational nature, it is not possible to make causal inferences between UF^NET^ intensity and outcomes. Second, we do not know precisely whether a UF^NET^ threshold > 25 ml/kg/day is associated with better outcomes, although our findings were robust to several sensitivity analyses. Third, our single-center study may not be generalizable to other ICU populations. Nevertheless, our study included patients typical of an academic medical center ICU population. Fourth, we were unable to distinguish whether patients received low-intensity UF^NET^ due to low prescription, failure to remove fluid (e.g., circuit downtime, trip to operating room, etc.) or other variations in practice with respect to fluid removal. Fifth, although the sensitivity analysis indicated that any unmeasured confounder would need to be highly prevalent and have an OR < 0.7 to mask a null association, it is possible that there may be more than one residual confounder and that it may not be a binary variable.

## Conclusion

In summary, among critically ill patients with ≥ 5% FO receiving RRT, our study found that UF^NET^ intensity > 25 ml/kg/day is associated with lower risk-adjusted 1-year mortality compared with < 20 ml/kg/day. Whether this association between UF^NET^ intensity > 25 ml/kg/day and lower mortality risk is just a marker for recovery or a mediator needs to be refuted or confirmed in future prospective randomized controlled trials.

## Additional file

Additional file 1: **S1.** Study population. **S2.** Determination of cumulative fluid balance. **S3.** Vasopressor standardization to norepinephrine equivalents. **S4.** Gray’s survival model. **S5.** Propensity score estimation and matching. **S6.** Quantitative bias sensitivity analysis of potential impact of an unmeasured confounder. **Figure S1.** Study population and analysis cohort. **Figure S2.** Association between intensity of net ultrafiltration and crude hospital mortality. **Figure S3.** Association between net ultrafiltration intensity and time to mortality using Gray’s model. **Figure S4.** Quantitative bias sensitivity analysis to assess the impact of an unmeasured confounder on mortality. **Table S1.** Cumulative fluid balance, mean arterial pressure and vasopressor dose for entire duration of RRT. **Table S2.** Association between net ultrafiltration intensity and 1-year risk-adjusted mortality. **Table S3.** Association between net ultrafiltration intensity and 1-year risk-adjusted mortality using net ulftrafiltration as a continuous variable. **Table S4.** Baseline characteristics by net ultrafiltration intensity after propensity matching (PDF 2022 kb)

## Abbreviations

AHR: Adjusted hazard ratio
AKI: Acute kidney injury
AOR: Adjusted odds ratio
APACHE: Acute Physiology and Chronic Health Evaluation
CRRT: Continuous renal replacement therapy
CVVH: Continuous venovenous hemofiltration
CVVHD: Continuous venovenous hemodialysis
CVVHDF: Continuous venovenous hemodiafiltration
FO: Fluid overload
IHD: Intermittent hemodialysis
MAP: Mean arterial pressure
RRT: Renal replacement therapy
SCUF: Slow continuous ultrafiltration
UF^NET^: Net ultrafiltration

## References

[1] Balakumar V, Murugan R, Sileanu FE, Palevsky P, Clermont G, Kellum JA (2017). Both positive and negative fluid balance may be associated with reduced long-term survival in the critically ill. Crit Care Med.

[2] Bellomo R, Cass A, Cole L, Finfer S, Gallagher M, Lee J, Lo S, McArthur C, McGuiness S (2012). An observational study fluid balance and patient outcomes in the randomized evaluation of normal vs. augmented level of replacement therapy trial. Crit Care Med.

[3] Vaara ST, Korhonen AM, Kaukonen KM, Nisula S, Inkinen O, Hoppu S, Laurila JJ, Mildh L, Reinikainen M, Lund V (2012). Fluid overload is associated with an increased risk for 90-day mortality in critically ill patients with renal replacement therapy: data from the prospective FINNAKI study. Crit Care.

[4] Bouchard J, Soroko SB, Chertow GM, Himmelfarb J, Ikizler TA, Paganini EP, Mehta RL, Program to Improve Care in Acute Renal Disease Study Group (2009). Fluid accumulation, survival and recovery of kidney function in critically ill patients with acute kidney injury. Kidney Int.

[5] KDIGO (2012). Clinical practice guideline for acute kidney injury. Kidney Int Suppl.

[6] Rosner MH, Ostermann M, Murugan R, Prowle JR, Ronco C, Kellum JA, Mythen MG, Shaw AD, ADQI XII Investigators Group (2014). Indications and management of mechanical fluid removal in critical illness. Br J Anaesth.

[7] Alwall N (1947). On the artificial kidney; apparatus for dialysis of the blood in vivo. Acta Med Scand.

[8] Flythe JE, Curhan GC, Brunelli SM (2013). Disentangling the ultrafiltration rate-mortality association: the respective roles of session length and weight gain. Clin J Am Soc Nephrol.

[9] Davies SJ, Brown EA, Reigel W, Clutterbuck E, Heimburger O, Diaz NV, Mellote GJ, Perez-Contreras J, Scanziani R, D’Auzac C (2006). What is the link between poor ultrafiltration and increased mortality in anuric patients on automated peritoneal dialysis? Analysis of data from EAPOS. Perit Dial Int.

[10] Burton JO, Jefferies HJ, Selby NM, McIntyre CW (2009). Hemodialysis-induced repetitive myocardial injury results in global and segmental reduction in systolic cardiac function. Clin J Am Soc Nephrol.

[11] Silversides JA, Pinto R, Kuint R, Wald R, Hladunewich MA, Lapinsky SE, Adhikari NK (2014). Fluid balance, intradialytic hypotension, and outcomes in critically ill patients undergoing renal replacement therapy: a cohort study. Crit Care.

[12] Flythe JE, Kimmel SE, Brunelli SM (2011). Rapid fluid removal during dialysis is associated with cardiovascular morbidity and mortality. Kidney Int.

[13] Movilli E, Gaggia P, Zubani R, Camerini C, Vizzardi V, Parrinello G, Savoldi S, Fischer MS, Londrino F, Cancarini G (2007). Association between high ultrafiltration rates and mortality in uraemic patients on regular haemodialysis. A 5-year prospective observational multicentre study. Nephrol Dial Transplant.

[14] Saran R, Bragg-Gresham JL, Levin NW, Twardowski ZJ, Wizemann V, Saito A, Kimata N, Gillespie BW, Combe C, Bommer J (2006). Longer treatment time and slower ultrafiltration in hemodialysis: associations with reduced mortality in the DOPPS. Kidney Int.

[15] Rewa OG, Villeneuve PM, Lachance P, Eurich DT, Stelfox HT, Gibney RTN, Hartling L, Featherstone R, Bagshaw SM (2017). Quality indicators of continuous renal replacement therapy (CRRT) care in critically ill patients: a systematic review. Intensive Care Med.

[16] Flythe JE, Assimon MM, Wenger JB, Wang L (2016). Ultrafiltration rates and the quality incentive program: proposed measure definitions and their potential dialysis facility implications. Clin J Am Soc Nephrol.

[17] Kellum JA, Murugan R (2016). Effects of non-severe acute kidney injury on clinical outcomes in critically ill patients. Crit Care.

[18] Liang KV, Sileanu FE, Clermont G, Murugan R, Pike F, Palevsky PM, Kellum JA (2016). Modality of RRT and recovery of kidney function after AKI in patients surviving to hospital discharge. Clin J Am Soc Nephrol.

[19] Neri M, Villa G, Garzotto F, Bagshaw S, Bellomo R, Cerda J, Ferrari F, Guggia S, Joannidis M, Kellum J (2016). Nomenclature for renal replacement therapy in acute kidney injury: basic principles. Crit Care.

[20] Hill ME, Rosenwaike I (2001). Social Security Administration's Death Master File: the completeness of death reporting at older ages. Soc Sec Bull.

[21] Saran R, Li Y, Robinson B, Ayanian J, Balkrishnan R, Bragg-Gresham J, Chen J, Cope E, Gipson D, He K (2015). US Renal Data System 2014 annual data report: epidemiology of kidney disease in the United States. Am J Kidney Dis.

[22] Kasal J, Jovanovic Z, Clermont G, Weissfeld LA, Kaplan V, Watson RS, Angus DC (2004). Comparison of cox and Gray’s survival models in severe sepsis. Crit Care Med.

[23] Valenta Z, Weissfeld L (2002). Estimation of the survival function for Gray's piecewise-constant time-varying coefficients model. Stat Med.

[24] Lash Timothy L., Fox Matthew P., Fink Aliza K. (2009). Applying Quantitative Bias Analysis to Epidemiologic Data.

[25] Lin DY, Psaty BM, Kronmal RA (1998). Assessing the sensitivity of regression results to unmeasured confounders in observational studies. Biometrics.

[26] Network VNARFT, Palevsky PM, Zhang JH, O'Connor TZ, Chertow GM, Crowley ST, Choudhury D, Finkel K, Kellum JA, Paganini E (2008). Intensity of renal support in critically ill patients with acute kidney injury. N Engl J Med.

[27] Bellomo R, Cass A, Cole L, Finfer S, Gallagher M, Lo S, McArthur C, McGuinness S, Myburgh J (2009). Intensity of continuous renal-replacement therapy in critically ill patients. N Engl J Med.

[28] O'Connor ME, Prowle JR (2015). Fluid overload. Crit Care Clin.

[29] O'Connor ME, Jones SL, Glassford NJ, Bellomo R, Prowle JR (2017). Defining fluid removal in the intensive care unit: a national and international survey of critical care practice. J Intensive Care Soc.

[30] Khanna A, English SW, Wang XS, Ham K, Tumlin J, Szerlip H, Busse LW, Altaweel L, Albertson TE, Mackey C (2017). Angiotensin II for the treatment of vasodilatory shock. N Engl J Med.

[31] Russell JA, Walley KR, Singer J, Gordon AC, Hebert PC, Cooper DJ, Holmes CL, Mehta S, Granton JT, Storms MM (2008). Vasopressin versus norepinephrine infusion in patients with septic shock. N Engl J Med.

[32] Vincent JL, Moreno R, Takala J, Willatts S, De Mendonca A, Bruining H, Reinhart CK, Suter PM, Thijs LG (1996). The SOFA (Sepsis-related Organ Failure Assessment) score to describe organ dysfunction/failure. On behalf of the Working Group on Sepsis-Related Problems of the European Society of Intensive Care Medicine. Intensive Care Med.

