# Supplementary material for: Net ultrafiltration intensity and mortality in critically ill patients with fluid overload
Source: Crit Care. 2018 Sep 24;22:223. doi: 10.1186/s13054-018-2163-1 (PMC6151928; doi:10.1186/s13054-018-2163-1)
Supplement: Supplementary file 1 — S1. Study population. S2. Determination of cumulative fluid balance. S3. Vasopressor standardization to norepinephrine equivalents. S4. Gray’s survival model. S5. Propensity score estimation and matching. S6. Quantitative bias sensitivity analysis of potential impact of an unmeasured confounder. Figure S1. Study population and analysis cohort. Figure S2. Association between intensity of net ultrafiltration and crude hospital mortality. Figure S3. Association between net ultrafiltration intensity and time to mortality using Gray’s model. Figure S4. Quantitative bias sensitivity analysis to assess the impact of an unmeasured confounder on mortality. Table S1. Cumulative fluid balance, mean arterial pressure and vasopressor dose for entire duration of RRT. Table S2. Association between net ultrafiltration intensity and 1-year risk-adjusted mortality. Table S3. Association between net ultrafiltration intensity and 1-year risk-adjusted mortality using net ulftrafiltration as a continuous variable. Table S4. Baseline characteristics by net ultrafiltration intensity after propensity matching (PDF 2022 kb) [file 13054_2018_2163_MOESM1_ESM.pdf]

Additional File 1

Net Ultrafiltration Intensity and Mortality in Critically Ill Patients with Fluid Overload

Raghavan Murugan, MD, MS, FRCP, FCCM, Vikram Balakumar, MD, Samantha J. Kerti, MS, Priyanka Priyanka, BAMS, MPH, Chung-Chou H. Chang, PhD, Gilles Clermont, MD, MSc, Rinaldo Bellomo MD, PhD, Paul M. Palevsky, MD,

John A. Kellum, MD, MCCM

Table of Contents

Online Data Supplement 1
S1: Study Population 3
S2: Determination of Cumulative Fluid Balance 4
S3: Vasopressor Standardization to Norepinephrine Equivalents 6
S4: Gray’s Survival Model 7
S5: Propensity Score Estimation and Matching 8
S6: Quantitative Bias Sensitivity Analysis of a Potential Impact of an Unmeasured Confounder 9
Figure E1 10
Figure E2 11
Figure E3 12
Figure E4 14
Table E1 16
Table E2 17
Table E3 18
Table E4 19
References 21

S1: Study Population

For each patient the following information were extracted: age, sex, primary diagnosis and patient comorbidities (coded according to the International Statistical Classification of Diseases and Related Health Problems, Ninth Revision [ICD-9]). For patients with multiple ICU admissions, the first ICU admission was considered as the reference point for our analyses. Severity of illness was computed from electronic abstraction of all physiologic variables comprising the Acute Physiologic and Chronic Health Evaluation (APACHE)-III score [1]. Because sepsis is underreported with ICD-9 codes, we defined "suspected sepsis" as the ordering of blood cultures and intravenous antibiotics within 24 hours of each other, as defined previously [2]. Following initiation of RRT, we extracted hourly mean arterial pressure (MAP) and vasopressor type and dose, and daily fluid balance for the duration of RRT.

We excluded patients with no available baseline hospital weight since fluid balance is expressed as a body weight percentage (Figure E1). We excluded patients discharged within 48 hours of ICU admission since most were either post-operative patients or in whom the critical illness resolved rapidly. We excluded those who died within 72 hours of ICU admission since any immediate death after ICU admission is more likely due to severity of illness. Since patients with end-stage renal disease and those with chronic kidney disease have different prognosis, we excluded patients on chronic dialysis, serum creatinine ≥3.5mgs/dl, and renal transplantation. We excluded patients with missing fluid balance and UF^{NET} data. Since the goal of this study is to examine the association between UF^{NET} intensity and mortality in patient with FO, we excluded patients with ≤5% FO and those who never received RRT.

S2: Determination of Cumulative Fluid Balance

We calculated cumulative fluid balance expressed as percentage of body weight for each patient prior to initiation of RRT and included only patients with ≥5% cumulative fluid balance as it was associated with long-term mortality in our prior work [3]. For each patient, we extracted input fluid including all intravenous and enteral fluids including resuscitation and maintenance fluids in the form of colloids and crystalloids, blood products, drug infusions, and enteral and parenteral nutrition. Output fluid included all body fluids (output from drains, rectal, orogastric and nasogastric output) including urine. We determined the cumulative FB expressed as percentage (%) from ICU admission until initiation of renal replacement therapy (RRT) using the following equation [3, 4]:

$$\text{Cumulative Fluid Balance (\%)} = \frac{\text{(Cumulative fluid input} - \text{fluid output) in litres X 100}}{\text{Hospital admission weight (kg)}}$$

For inclusion in the study, we identified patients who only had cumulative fluid balance ≥5% of body weight from the time of ICU admission prior to initiation of either intermittent hemodialysis (IHD) or continuous renal replacement therapy (CRRT). We used ≥5% cumulative fluid balance as a threshold as it was associated with both short- and long-term mortality in our prior work [3]. We excluded patients from our analysis if they had a cumulative fluid balance <5% of body weight before initiation of RRT. In order to determine the patient's cumulative fluid balance after the initiation of RRT, we used the following approach. For patients receiving CRRT, daily cumulative fluid balance was calculated as input minus output excluding the net volume of fluid removed during CRRT (*i.e.,* UF^{NET}, which is the exposure variable). For patients receiving IHD,

cumulative fluid balance including the inter-dialytic period was calculated as daily input minus output excluding UF^{NET} during each IHD session.

S3: Vasopressor Standardization to Norepinephrine Equivalents

All vasopressors were standardized according to the following conversion scale below. The conversion scale was developed based on the cardiovascular Sequential Organ Failure Assessment score and the medical literature [5, 6]. Vasopressin equivalence to norepinephrine was developed with the use of the Vasopressin and Septic Shock Trial data set [7].

Drug	Dose	Norepinephrine Equivalent
Epinephrine	0.1 mcg/kg/min	0.1 mcg/kg/min
Norepinephrine	0.1 mcg/kg/min	0.1 mcg/kg/min
Dopamine	15 mcg/kg/min	0.1 mcg/kg/min
Phenylephrine	1.0 mcg/kg/min	0.1 mcg/kg/min
Vasopressin	0.04 U/min	0.1 mcg/kg/min

S4: Gray’s Survival Model

The Cox multivariable regression model relies on the assumption that the proportionality of hazards remains constant over the length of duration that it’s used to estimate the conditional hazard rate. However, in most real clinical scenarios, especially in acute illness, wherein multiple clinical factors affect a patient simultaneously, this may not hold true. To address this issue, models that allow for non-proportionality of the conditional hazards by introducing covariate effects have been proposed. The Gray's model, proposed by Gray [8] is one such model that employs products of the covariates of interest with the spline functions of time [8].

The advantage of the Gray's model is that it retains most of the mathematical simplicity of the Cox model since the proportional hazards assumption is only required for each of the time intervals between the successive knots (*i.e.,* the time points within the duration over which treatment effects are studied) [9]. Gray’s model may therefore be viewed as a piecewise Cox proportional hazards model for the conditional hazard rate. We used Gray’s model in this manuscript, as well as prior work [3, 10], for two reasons: a.) Cox models failed proportionality assumptions for several covariates, and b.) to assess the variation in AHRs associated with UF^{NET} intensity on mortality over time.

S5: Propensity Score Estimation and Matching

We constructed a propensity score to account for indication bias associated with UF^{NET} using multinomial logistic regression with UF^{NET} intensity categorized as low (≤ 25ml/kg/day) and high (>25ml/kg/day) as an outcome. The variables used in the model for propensity score estimation included age, sex, race, body mass index, admission under surgical service, admission for liver transplantation, baseline estimated glomerular filtration rate (eGFR), APACHE-III score, mechanical ventilation, suspected sepsis, first RRT modality, cumulative fluid overload before initiation of RRT, total duration of RRT, time-to-initiation of RRT, mean arterial pressure on day 1 of RRT, cumulative vasopressor dose and cumulative fluid balance after initiation of RRT. We then matched the low (≤25ml/kg/day) with that of high intensity UF^{NET} (>25ml/kg/day) using propensity scores on 1:1 basis without replacement creating 258-matched pairs. Matches were created without replacement using computational geometry based on distance between propensity scores. The matching was based on a 1:1 ratio along with a distance set by a caliper of 0.025. The analysis was carried out using source code and SAS macros that are available online [11].

S6: Quantitative Bias Sensitivity Analysis of a Potential Impact of an Unmeasured Confounder

To estimate the potential impact of an unmeasured confounder [12], we made the following assumptions: 1) only one unmeasured confounder was present (or a combination of confounders that can be described as one), 2) the unmeasured confounder is binary, 3) the unmeasured confounder is independent of measured confounders, and 4) the exposure UF^{NET} intensity is not an effect modifier for the unmeasured confounder's effect on outcome.

The impact of the unmeasured confounder was determined by the following: 1) the prevalence of the unmeasured confounder in the exposed (*i.e.,* high UF^{NET} group) vs. the unexposed group (*i.e.,* low intensity UF^{NET} group) and 2) the association between the unmeasured confounder and mortality, independent of the measured confounders, expressed as an odds ratio (*e.g.,* an OR=0.8 indicates that the odds of death is 20% lower than those with the unmeasured confounder compared to those without the unmeasured confounder). We independently varied these 2 parameters to assess their influence on the adjusted odds ratio for high intensity UF^{NET} on mortality and present the results in graphical form.

Figure E1

Study Population and Analysis Cohort

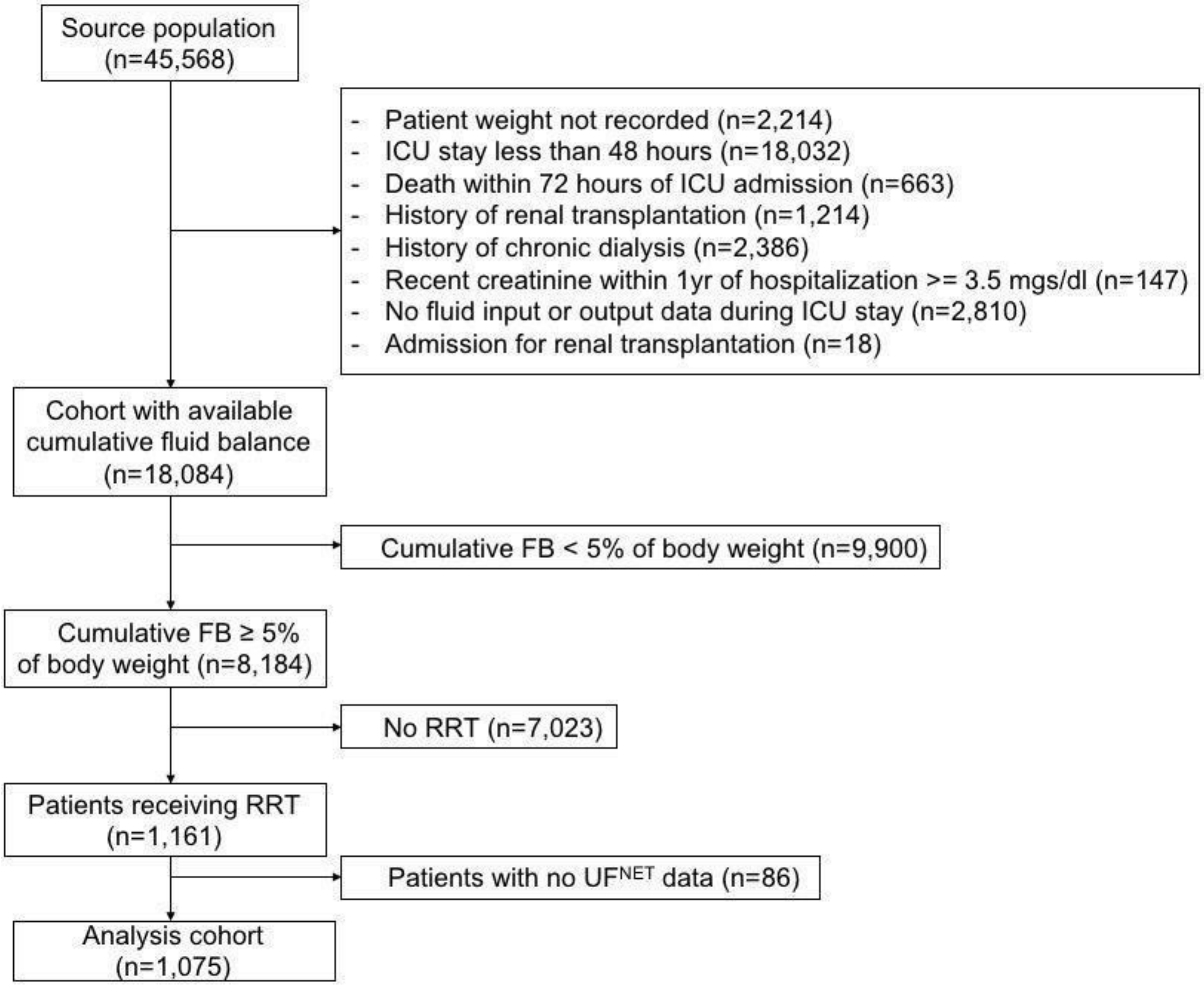

Figure E2

Association Between Intensity of Net Ultrafiltration and Crude Hospital Mortality

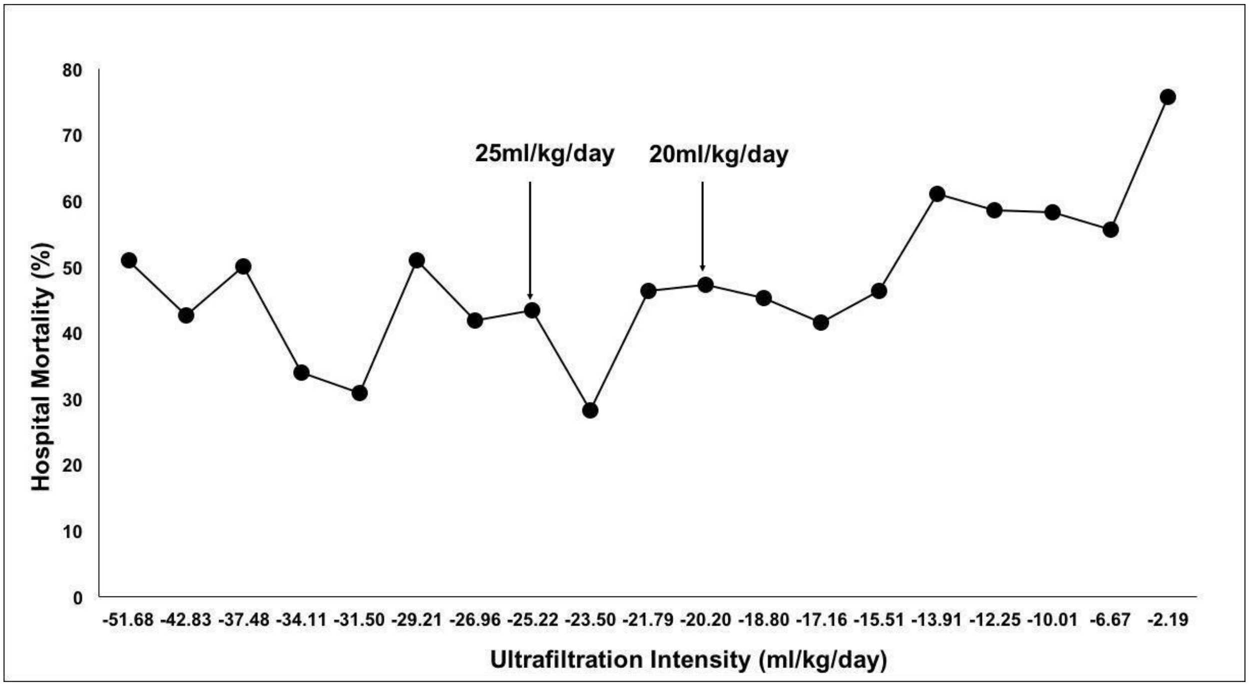

X -axis represents the UF^{NET} expressed in ml/kg/day. Y-axis corresponds to the crude hospital mortality. The first arrow on the left represents the point on the distribution curve that corresponds to UF^{NET} 25ml/kg/day and the second arrow on the right corresponds to the point on the distribution curve that corresponds to UF^{NET} 20ml/kg/day.

Figure E3

Association between Net Ultrafiltration Intensity and Time-to-Mortality Using Grays model

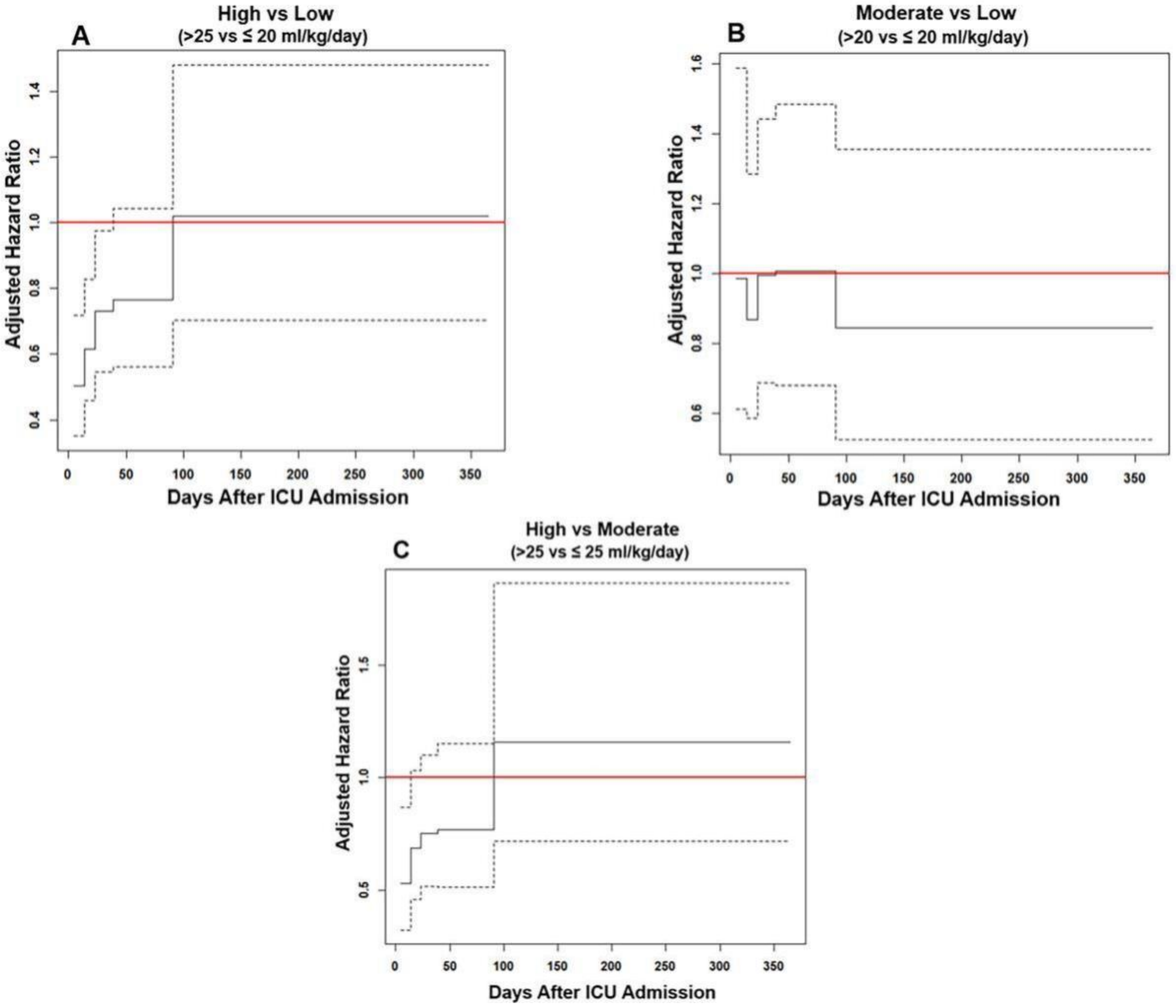

Figure shows varying adjusted hazard ratios with 95% CIs for risk of death over 365 days estimated from Gray's model using four time nodes and five intervals. A hazard ratio <1 suggests that UF^{NET} is associated with lower mortality and a hazard ratio >1 suggests higher mortality. Adjusted for age, sex, race, body mass index, history of liver disease and sequela from liver disease, admission for liver transplantation, admission for surgery, baseline glomerular filtration rate, APACHE-III score, presence of sepsis, use of mechanical ventilation, percentage of FO before initiation of RRT, oliguria before initiation of RRT, time-to-initiation of RRT from ICU admission, mean arterial pressure

on first day of RRT initiation, cumulative vasopressor dose and cumulative fluid balance during RRT, first RRT modality and duration of RRT.

A. High UF^{NET} (>25ml/kg/day) as compared with low UF^{NET} (≤20ml/kg/day), was associated with decreased risk for death in the first 39 days after ICU admission, however subsequently, there was no association with mortality.

B. Whereas, moderate UF^{NET} (>20-≤25ml/kg/day), as compared with low UF^{NET} (≤20ml/kg/day), was not associated with mortality.

C. High UF^{NET} (>25ml/kg/day), compared with moderate UF^{NET} (>20-≤25ml/kg/day), was associated with decreased risk of death only up to 15 days after ICU admission.

Figure E4
Quantitative Bias Sensitivity Analysis to Assess the Impact of an Unmeasured Confounder on Mortality

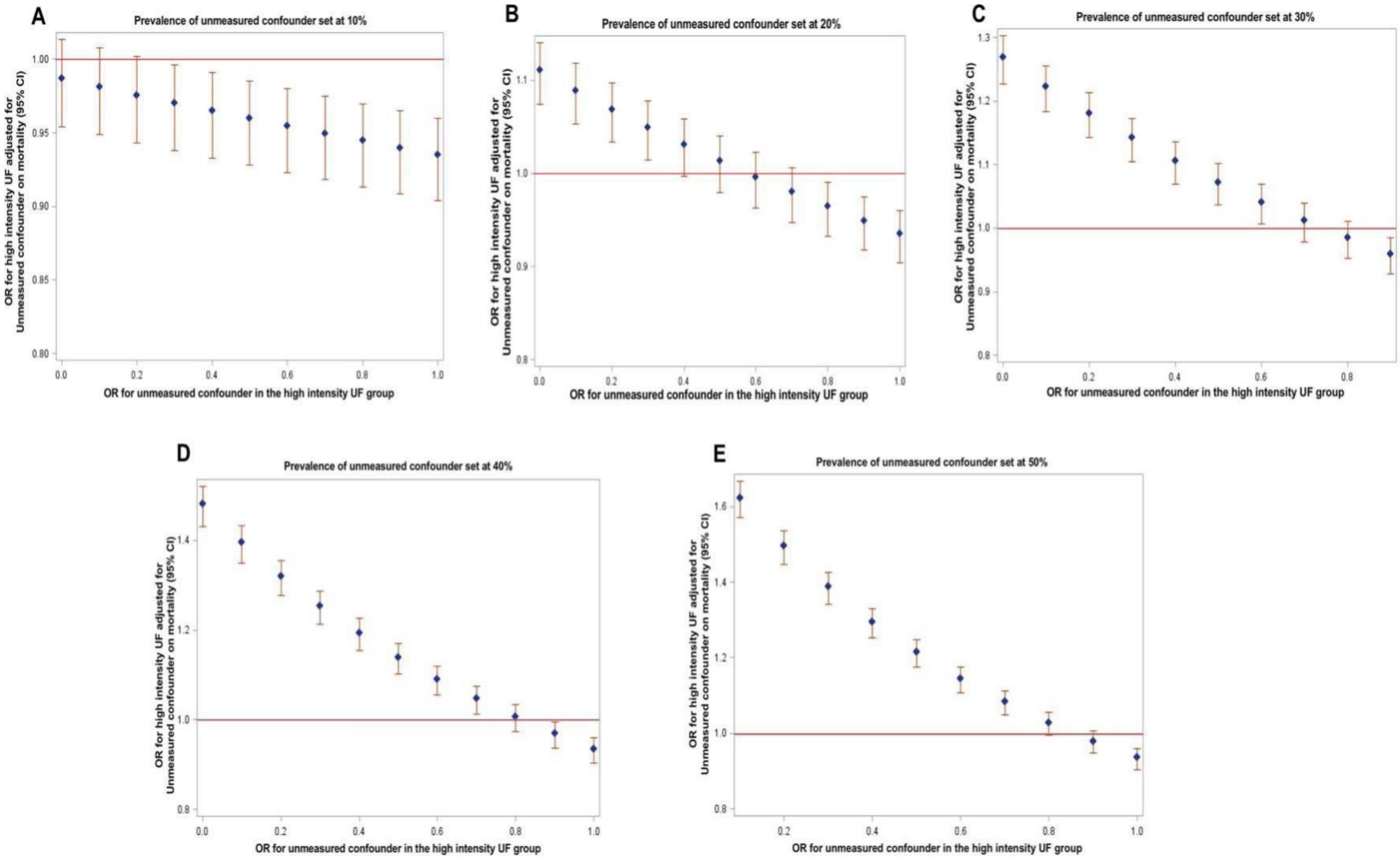

Quantitative bias sensitivity analysis of a hypothetical unmeasured confounder.[12] The above plot shows the strength of a hypothetical confounder (X axis, univariate odds ratios of confounder in the high intensity UF^{NET} group on 1-year mortality) versus the odds ratio for 1-year mortality from adjusted model for high intensity UF^{NET}, if the hypothetical confounder was included (Y axis). Panels A, B, C, D, and E, correspond to varying prevalence of unmeasured confounder among patients in the high intensity UF^{NET} group of 10%, 20%, 30%, 40% and 50%, respectively, compared with patients in the low intensity UF^{NET} group.

To abrogate the odds ratio for high intensity UF^{NET} from our primary analysis (*i.e.,* OR=0.61, 95% CI, 0.41-0.93), the hypothetical unmeasured confounder must be at least twice as common (prevalence of 20% or more, panels B, C, D, and E) among patients with high intensity U^{FNET}, and have an odds ratio for 1-year mortality that is lower than 0.7 (*i.e.,* associated with more than 30% lower risk of death). Stronger confounders are required if the prevalence among patients who received high intensity UF^{NET} is lower than 10%.

Table E1

Cumulative Fluid Balance, Mean Arterial Pressure and Vasopressor Dose For Entire Duration of RRT

Characteristic	N (%)			P value
	≤20 ml/kg/day (N=475)	>20 to ≤25 ml/kg/day (n=166)	>25 ml/kg/day (n=434)	
Cumulative FB excluding UF^{NET}, L, Median(IQR)				
CRRT	8.8 (3.4 – 20.5)	11 (5.7 – 24.3)	14.6 (5.8 – 30.7)	0.002
IHD	3.1 (0.9 – 15.8)	10.6 (4.3 – 41)	9.8 (3.3 – 25.1)	0.002
Both CRRT and IHD	36.5 (17.6 – 61.6)	37.3 (18.6 – 63.6)	28.6 (15.4 – 54.5)	0.12
MAP, mmHg, Mean(SD)				
CRRT	71.4 (0.78)	72 (1.23)	77.6 (0.88)	<0.001
IHD	84 (1.57)	82.4 (1.79)	84 (1.06)	0.63
Both CRRT and IHD	77.3 (0.84)	79.7 (1.00)	80.2 (0.79)	0.07
Cumulative vasopressor dose, NE, Median(IQR)				
CRRT	22.6 (9.7 – 47.3)	20.4 (7.3 – 45.1)	18.6 (5.3 – 43.3)	0.25
IHD	0.67 (0 – 3.9)	0.001 (0 – 1.7)	0.14 (0 – 2.8)	0.5
Both CRRT and IHD	14.9 (4.97 – 28.7)	12.6 (2.6 – 26.8)	12.4 (3.8 – 24.5)	0.45

MAP, Mean Arterial Pressure; CRRT, Continuous renal replacement therapy; IHD, Intermittent hemodialysis; FB, Fluid balance; NE, nor-epinephrine equivalent.

Table E2

Association Between UF^{NET} Intensity and 1-year Risk-Adjusted Mortality

Covariates	Unadjusted Odds Ratio (95%CI)	P Value	Adjusted Odds Ratio (95%CI)	P value
Moderate vs. low intensity UF^{NET} (reference)	0.65 (0.42 – 0.94)	0.024	0.81 (0.48 – 1.35)	0.41
High vs. low intensity UF^{NET} (reference)	0.64 (0.49 – 0.85)	0.002	0.61 (0.41 – 0.93)	0.02
Age	1.04 (1.03 – 1.05)	<0.001	1.049 (1.03 – 1.06)	<0.001
Male vs female	1.10 (0.85 – 1.42)	0.45	1.25 (0.88 – 1.78)	0.21
Race				
Other vs. white	1.37 (0.94 – 2.00)	0.098	1.94 (1.11 – 3.40)	0.02
Black vs. white	1.12 (0.67 – 1.85)	0.67	0.68 (0.33 – 1.40)	0.30
Body mass index	0.99 (0.98 – 1.01)	0.91	0.99 (0.97 – 1.01)	0.35
History of sequela of liver disease	0.71 (0.54 – 0.95)	0.02	1.31 (0.61 – 2.80)	0.5
History of liver disease	0.80 (0.61 – 1.05)	0.102	1.79 (0.83 – 3.85)	0.14
Admission for liver transplant	0.24 (0.17 – 0.33)	<0.001	0.16 (0.90 – 0.31)	<0.001
Surgical admission	0.36 (0.26 – 0.49)	<0.001	0.31 (0.19 – 0.49)	<0.001
Chronic kidney disease				
Stage 5 vs 1	1.26 (0.51 – 3.11)	0.62	0.39 (0.12 – 1.28)	0.12
Stage 4 vs 1	1.74 (0.98 – 3.09)	0.057	0.97 (0.44 – 2.13)	0.93
Stage 3 vs 1	1.30 (0.88 – 1.91)	0.19	0.77 (0.44 – 1.35)	0.35
Stage 2 vs 1	1.34 (0.97 – 1.84)	0.07	0.86 (0.54 – 1.38)	0.54
APACHE III score	1.00 (0.99 – 1.00)	0.92	1.01 (1.00 – 1.01)	0.04
Mechanical Ventilation	0.67 (0.50 – 0.91)	0.01	0.75 (0.46 – 1.23)	0.26
Sepsis	0.96 (0.73 – 1.27)	0.8	0.84 (0.56 – 1.26)	0.4
Oliguria	1.06 (0.46 – 2.45)	0.88	1.14 (0.37 – 3.56)	0.82
Cumulative fluid overload before RRT	1.01 (1.00 – 1.01)	0.043	1.01 (0.99 – 1.02)	0.12
No. of days from ICU admission to RRT initiation	1.02 (1.00 – 1.03)	0.005	1.03 (1.01 – 1.06)	0.013
First RRT Modality				
CRRT vs. IHD	1.20 (0.91 – 1.58)	0.19	0.95 (0.63 – 1.44)	0.80
Mean arterial pressure on day 1 of RRT	0.96 (0.95 – 0.98)	<0.001	0.98 (0.96 – 0.99)	0.005
Cumulative vasopressor dose during RRT	1.01 (1.01 – 1.02)	<0.001	1.01 (1.00 – 1.02)	0.004
Cumulative fluid balance during RRT	1.00 (1.00 – 1.00)	0.45	1.00 (1.00 – 1.00)	0.046
RRT duration	1.001 (0.99 – 1.01)	0.83	0.99 (0.97 – 1.02)	0.70

Table E3

Association Between UF^{NET} Intensity and 1-year Risk-Adjusted Mortality using UF^{NET} as a Continuous Variable

Covariates	Unadjusted Odds Ratio (95%CI)	P Value	Adjusted Odds Ratio (95%CI)	P value
UF^{NET} per ml/kg/day	0.99 (0.98 – 0.99)	<0.001	0.98 (0.97 – 0.99)	0.005
Age	1.04 (1.03 – 1.05)	<0.001	1.05 (1.03 – 1.06)	<0.001
Male vs female	1.10 (0.85 – 1.42)	0.45	1.32 (0.93 – 1.89)	0.12
Race				
Other vs. white	1.40 (0.94 – 2.00)	0.10	1.96 (1.12 – 3.43)	0.02
Black vs. white	1.12 (0.67 – 1.85)	0.7	0.64 (0.31 – 1.31)	0.22
Body mass index	0.10 (0.98 – 1.01)	0.91	0.99 (0.97 – 1.01)	0.25
History of sequela of liver disease	0.71 (0.54 – 0.95)	0.02	1.30 (0.61 – 2.8)	0.50
History of liver disease	0.80 (0.61 – 1.05)	0.10	1.82 (0.85 – 3.89)	0.13
Admission for liver transplant	0.24 (0.20 – 0.33)	<0.001	0.16 (0.08 – 0.30)	<0.001
Surgical admission	0.36 (0.26 – 0.49)	<0.001	0.31 (0.19 – 0.50)	<0.001
Chronic kidney disease				
Stage 5 vs 1	1.26 (0.51 – 3.12)	0.62	0.41 (0.12 – 1.32)	0.13
Stage 4 vs 1	1.74 (0.98 – 3.1)	0.057	0.95 (0.43 – 2.1)	0.90
Stage 3 vs 1	1.30 (0.89 –1.92)	0.19	0.75 (0.43 –1.32)	0.31
Stage 2 vs 1	1.34 (0.97 – 1.84)	0.07	0.85 (0.54 –1.36)	0.50
APACHE III score	1.00 (0.99 – 1.00)	0.92	1.01 (1.00 – 1.01)	0.052
Mechanical Ventilation	0.67 (0.50 – 0.91)	0.011	0.78 (0.48 –1.27)	0.32
Sepsis	0.96 (0.73 – 1.27)	0.78	0.81 (0.54 – 1.23)	0.33
Oliguria	1.06 (0.46 – 2.45)	0.88	1.20 (0.39 – 3.74)	0.75
Cumulative fluid overload before RRT	1.01 (1.00 – 1.01)	0.043	1.01 (0.99 – 1.02)	0.12
No. of days from ICU admission to RRT initiation	1.02 (1.00 – 1.03)	0.005	1.03 (1.01 – 1.06)	0.01
First RRT Modality				
CRRT vs. IHD	1.20 (0.91 – 1.58)	0.20	0.97 (0.64 – 1.47)	0.90
Mean arterial pressure on day 1 of RRT	0.97 (0.95 – 0.98)	<0.001	0.98 (0.96 – 0.99)	0.007
Cumulative vasopressor dose during RRT	1.01 (1.01 – 1.02)	<0.001	1.01 (1.00 – 1.02)	0.005
Cumulative fluid balance during RRT	1.00 (1.00 – 1.00)	0.45	1.00 (1.00 – 1.00)	0.04
RRT duration	1.00 (0.99 – 1.01)	0.83	0.99 (0.99 – 1.03)	0.71

Table E4

Baseline Characteristics by Intensity of UF^{NET} after Propensity Score Matching

Characteristic	No(%) ≤25mls/kg/day (N=258)	>25mls/kg/day (N=258)	P value
Age, yrs, median (IQR)	60 (52 – 71)	59 (49 – 72)	0.51
Male	144 (55.8)	145 (56.2)	0.93
Race			
Caucasian	208 (80.6)	205 (79.5)	
African-American	14 (5.4)	18 (7)	0.76
Other	36 (13.9)	35 (13.6)	
BMI, kg/m2 median (IQR)	25.8 (23.3 – 29.8)	25.8 (22.7 – 30.3)	0.75
APACHE-III score, Median(IQR)[a]	97 (69 –121)	92 (72 – 117)	0.85
Baseline eGFR (mL/min/1.73m^2)			
>90	50 (19.4)	47 (18.2)	
60 - 90	132 (51.2)	134 (51.9)	
30 - 60	48 (18.6)	50 (19.4)	0.98
15 - 30	22 (8.5)	20 (7.7)	
<15	6 (2.3)	7 (2.7)	
Admission for liver transplant	37(14.3)	41 (15.9)	0.62
Surgical admission	184 (71.3)	185 (71.7)	0.92
Mechanical Ventilation [a]	194 (75.2)	198 (76.7)	0.70
Sepsis [a]	79 (30.6)	80 (31)	0.92
First RRT Modality			
CRRT	181 (70.2)	182 (70.5)	0.92
IHD	77 (29.8)	76 (29.5)	0.92
Cumulative fluid overload before RRT, % of body weight, median(IQR)	17.5 (11.2 – 27.6)	17.87 (11.7 – 28.1)	0.31
RRT duration, days median(IQR)	5.8 (2.1 – 12)	6.85 (3.4– 11.6)	0.06
No. of days from ICU admission to RRT initiation, days, median(IQR)	5 (2 – 11)	5 (2 – 11)	0.59
MAP on day 1 of RRT, mmHg, Mean(SD)[b]	75.7 (68.8 – 84.2)	77.57 (70.5 – 85.7)	0.26
Cumulative vasopressor dose during RRT, NE, median(IQR) [c]	13.3 (2.2 – 35.3)	8.3 (0.8 – 32)	0.02
Cumulative FB excluding UF^{NET} for duration of RRT, L, median(IQR)	15.6 (5.5 – 37.7)	19.9 (8.0 – 39.2)	0.15

Abbreviations: IQR, Interquartile range; BMI, body mass index; APACHE, Acute physiology and chronic health evaluation; eGFR, estimated glomerular filtration; RRT, renal replacement therapy; CRRT, continuous renal replacement therapy; IHD, intermittent hemodialysis; MAP, mean arterial pressure; NE, nor-epinephrine equivalents; FB, fluid balance.

Patients were matched using propensity scores for age, sex, race, body mass index, Acute physiology and chronic health evaluation (APACHE)-III score, baseline estimated glomerular filtration, admission for liver transplantation, admission to surgical service, mechanical ventilation, sepsis, first RRT modality, fluid balance prior to initiation of RRT, RRT duration, and time to initiation of RRT from ICU admission mean arterial pressure on first day of RRT initiation, cumulative vasopressor dose, cumulative fluid balance during RRT.

[a] At ICU admission

[b] On the day 1 of renal replacement therapy

[c] All vasopressors were standardized in terms of nor-epinephrine equivalents (Supplemental Table S3)[5-7].

Additional References

1. Knaus WA, Wagner DP, Draper EA, Zimmerman JE, Bergner M, Bastos PG, Sirio CA, Murphy DJ, Lotring T, Damiano A *et al*: The APACHE III prognostic system. Risk prediction of hospital mortality for critically ill hospitalized adults. *Chest* 1991, 100(6):1619-1636.
2. Rhodes A, Evans LE, Alhazzani W, Levy MM, Antonelli M, Ferrer R, Kumar A, Sevransky JE, Sprung CL, Nunnally ME *et al*: Surviving Sepsis Campaign: International Guidelines for Management of Sepsis and Septic Shock: 2016. *Crit Care Med* 2017, 45(3):486-552.
3. Balakumar V, Murugan R, Sileanu FE, Palevsky P, Clermont G, Kellum JA: Both Positive and Negative Fluid Balance May Be Associated With Reduced Long-Term Survival in the Critically Ill. *Crit Care Med* 2017, 45(8):e749-e757.
4. Selewski DT, Cornell TT, Lombel RM, Blatt NB, Han YY, Mottes T, Kommareddi M, Kershaw DB, Shanley TP, Heung M: Weight-based determination of fluid overload status and mortality in pediatric intensive care unit patients requiring continuous renal replacement therapy. *Intensive Care Med* 2011, 37(7):1166-1173.
5. Vincent JL, Moreno R, Takala J, Willatts S, De Mendonca A, Bruining H, Reinhart CK, Suter PM, Thijs LG: The SOFA (Sepsis-related Organ Failure Assessment) score to describe organ dysfunction/failure. On behalf of the Working Group on Sepsis-Related Problems of the European Society of Intensive Care Medicine. *Intensive Care Med* 1996, 22(7):707-710.
6. Khanna A, English SW, Wang XS, Ham K, Tumlin J, Szerlip H, Busse LW, Altaweel L, Albertson TE, Mackey C *et al*: Angiotensin II for the Treatment of Vasodilatory Shock. *N Engl J Med* 2017, 377(5):419-430.
7. Russell JA, Walley KR, Singer J, Gordon AC, Hebert PC, Cooper DJ, Holmes CL, Mehta S, Granton JT, Storms MM *et al*: Vasopressin versus norepinephrine infusion in patients with septic shock. *N Engl J Med* 2008, 358(9):877-887.
8. Kasal J, Jovanovic Z, Clermont G, Weissfeld LA, Kaplan V, Watson RS, Angus DC: Comparison of Cox and Gray's survival models in severe sepsis. *Crit Care Med* 2004, 32(3):700-707.

9. Valenta Z, Weissfeld L: Estimation of the survival function for Gray's piecewise-constant time-varying coefficients model. *Statistics in Medicine* 2002, 21(5):717-727.
10. Rimmele T, Karajala V, Murugan R, Kellum JA: Use of loop diuretics in the critically ill. *Contrib Nephrol* 2010, 165:219-225.
11. Rassen J, Doherty M, Huang W, Schneeweiss S: Pharmacoepidemiology Toolbox. Boston, MA. In.; 2014.
12. Lash TF, MP; Fink, AK: Applying quantitative bias analysis to epidemiologic data. *New York: Springer-Verlag*.
